# Sustained complete response of advanced hepatocellular carcinoma with metronomic capecitabine: a report of three cases

**DOI:** 10.1186/s40880-018-0312-1

**Published:** 2018-06-26

**Authors:** Giovanni Brandi, Michela Venturi, Stefania De Lorenzo, Francesca Garuti, Giorgio Frega, Andrea Palloni, Ingrid Garajovà, Francesca Abbati, Gioconda Saccoccio, Rita Golfieri, Maria Abbondanza Pantaleo, Maria Aurelia Barbera

**Affiliations:** 1 Department of Experimental, Diagnostic and Specialty Medicine, St. Orsola-Malpighi Hospital, Haematological and Oncological Institute, University of Bologna, 9 Massarenti street, 40138 Bologna, Italy; 2 Department of Medical and Surgical Sciences, St. Orsola-Malpighi Hospital, University of Bologna, 40138 Bologna, Italy; 3 Department of Medicine, Bologna Health Authority, 40139 Bologna, Italy; 4 Department of Digestive Diseases and Internal Medicine, St. Orsola-Malpighi Hospital, University of Bologna, 40138 Bologna, Italy

**Keywords:** Hepatocellular carcinoma, Capecitabine, Metronomic capecitabine, Complete response, Sorafenib

## Abstract

**Background:**

Hepatocellular carcinoma (HCC) is one of the most frequent causes of cancer-related death. Sorafenib, a multitarget angiogenesis inhibitor, is an approved frontline treatment for advanced HCC in Western countries, although a complete response (CR) to treatment is infrequently reported. Capecitabine, an oral fluoropyrimidine, has been shown to be effect in both treatment-naïve patients and those previously treated with sorafenib. To date, however, only one case of sustained CR to metronomic capecitabine has been reported.

**Case presentation:**

We describe three cases of advanced HCC treated with metronomic capecitabine where a CR was obtained. In the first case, capecitabine was administered as first line therapy; in the second case, capecitabine was used after intolerance to sorafenib; while in the third case, capecitabine was administered after sorafenib failure.

**Conclusion:**

Capecitabine is a potentially important treatment option for patients with advanced HCC and may even represent a cure in certain cases.

## Background

Hepatocellular carcinoma (HCC) is a major challenge for oncologists, being the fifth most common cancer in men and the seventh in women, representing the third most frequent cause of cancer death worldwide [1]. Treatment strategies for HCC depend on both tumour stage and liver disease status.

In patients with advanced HCC, or where surgical or locoregional treatment is contraindicated because of underlying cirrhosis or patient characteristics, medical therapy becomes the only strategy [2]. Sorafenib, a multikinase inhibitor, was the first molecular targeted therapy to demonstrate a survival benefit [3], and the only globally approved frontline treatment for advanced HCC. The efficacy of sorafenib has been demonstrated in two randomized, double-blinded, placebo-controlled phase III clinical trials: the Sorafenib HCC Assessment Randomized Protocol (SHARP) [3] and the Asia-Pacific [4] trials. Sorafenib is considered moderately effective in HCC treatment, with a less than 3-month increase in overall survival (OS) in comparison with placebo [3]. Cases of complete response (CR) to sorafenib have been reported in the literature [5], but no data on the duration of the CR are available.

Capecitabine is an oral fluoropyrimidine carbamate that acts as a 5-fluorouracil (5-FU) pro-drug to mimic the continuous infusion of 5-FU. It is globally approved for the treatments of colon, gastric, and breast cancer.

Capecitabine administration according to a standard (or reduced standard) schedule has been evaluated in the postoperative adjuvant setting after curative HCC resection [6], in advanced disease [7], and in recurrent HCC after liver transplantation [8]. In a small randomized controlled trial among patients with HCC, 30 controls treated with best supportive care (BSC) were compared with 30 subjects who received 2 weeks of capecitabine at 1000 mg/m^2^, twice a day, followed by 1 week without treatment for a total of 4–6 cycles [6]. The data showed that adjuvant therapy with capecitabine was well tolerated, postponed and reduced the risk of HCC recurrence, and was likely to improve postoperative survival.

In a single-centre phase II, open-label trial, 52 patients with advanced HCC who had not received previous systemic treatment were randomly assigned to receive either sorafenib (at a dose of 400 mg twice daily) or capecitabine to a modified standard schedule (1000 mg/m^2^ twice daily on days 1–14) [7]. Median OS was 7.05 months in the sorafenib group and 5.07 months in the capecitabine group. Thus, capecitabine at the modified dosing schedule was found to be inferior to sorafenib with respect to OS [7]. However, when capecitabine was administered according to a metronomic schedule, varying results were reported [9, 10].

Metronomic therapy is defined as the continuous administration of a lower dose of cytotoxic drug, without treatment pauses [11]. While standard chemotherapy exerts an antiproliferative effect, metronomic chemotherapy functions to inhibit angiogenesis by directly disrupting endothelial cell proliferation, up-regulating antiangiogenic factors such as thrombospondin-1 (TSP-1) and angiostatin, and down-regulating of angiogenic factors such as vascular endothelial growth factor (VEGF), basic fibroblast growth factor (b-FGF), and hypoxia-inducible factor-1 (HIF-1) [12]. Metronomic chemotherapy also inhibits vascular development by reducing the number and viability of circulating endothelial progenitor cells from bone marrow [13]. In addition, metronomic chemotherapy stimulates the immune response by reducing regulatory T cells and promoting dendritic cell maturation [14, 15]. Low-dose regimens of chemotherapy can also induce senescence in tumours through an antiproliferative response [16]. The use of metronomic treatment is becoming more frequent for the treatment of various cancers, owing to its antiangiogenic properties and tolerability. However, the effects of metronomic chemotherapy appear to vary according to solid tumour type.

Metronomic chemotherapy has been evaluated only to a limited extent for the treatment of advanced HCC. Hsu et al. [17] conducted a phase II study to evaluate the efficacy and safety sorafenib (400 mg twice daily) in combination with metronomic tegafur/uracil (UFT) (125 mg/m^2^ based on tegafur twice daily) in 53 patients with HCC and a Child–Pugh class A score. Median progression-free survival was 3.7 months, and median survival was 7.4 months. The use of metronomic chemotherapy and sorafenib was shown to augment antitumor efficacy without a high incidence of severe side effects.

Metronomic capecitabine for advanced HCC, in first line treatment, has been evaluated in a phase II study, and was shown to be well tolerated and associated with an OS of 14.47 months, further emphasized by a propensity analysis comparing treated subjects with matched controls [9]. Sustained CR to capecitabine was also reported [18]. In the present case report, we describe three cases of sustained CR following treatment with metronomic capecitabine in patients with advanced HCC. Informed consent to the treatment was obtained from the patients.

## Case presentation

### Case 1

An 84-year old man with chronic hepatitis C and liver cirrhosis was referred to our outpatient clinic for the evaluation of HCC, previously treated with transarterial chemoembolization (TACE) and radiofrequency ablation (RFA). Following disease relapse, a wedge resection of two nodules in hepatic segments VI and VII was performed in December 2008. Histological examination confirmed HCC grade III (Edmondson scoring), with necrosis and microscopic vascular thrombosis.

**Fig. 1:**
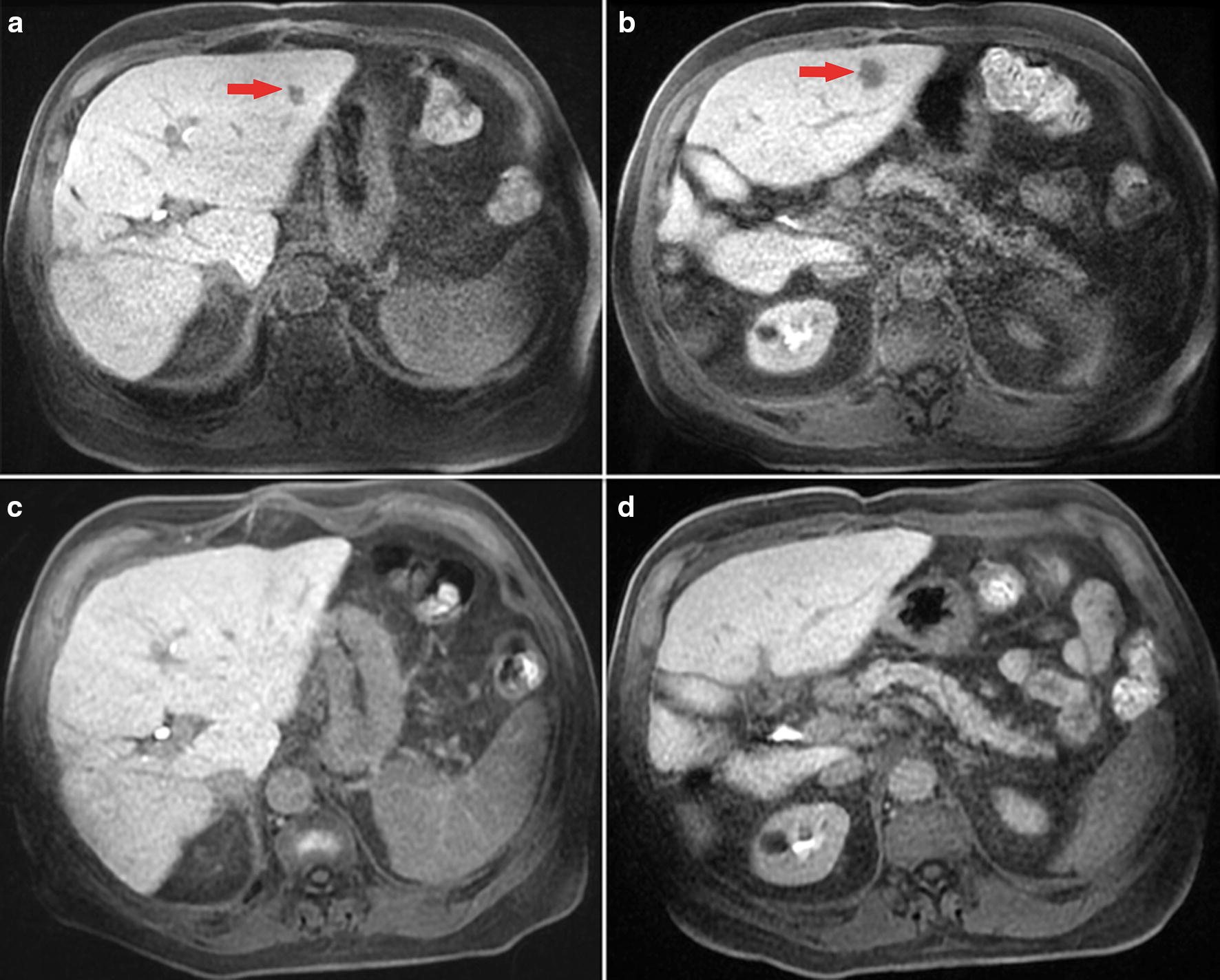
Lesions in case 1. Gd-EOB-DTPA-enhanced MR images showing, at baseline acquisition, hepatobiliary phase 4 millimetric lesions: a 10 mm hepatocellular carcinoma (HCC) nodule in segment II (**a**), a 14 mm HCC nodule in segment III (**b**), and a millimetric lesion at segments V and I. Gd-EOB-DTPA-enhanced MR follow-up after 89 months since starting systemic treatment with metronomic capecitabine shows a stable complete response, with the absence of both HCC nodules (**c**, **d**)

In September 2009, magnetic resonance imaging (MRI) showed millimetric disease relapse in hepatic segments V, II, III, and I, and a 21 × 9 mm adenopathy at the hepatic hilum (Fig. 1a, b). A new resection was scheduled but was not carried out following the detection by intra-operative ultrasound (US) of a right portal branch neoplastic thrombosis. In December 2009, serum alpha-fetoprotein (AFP) was 1504 ng/mL. In January 2010, as a consequence of disease metastasis, systemic treatment with metronomic capecitabine (500 mg twice daily) was continuously administered according to a previously described protocol [6]. The therapy was well tolerated. After 1 month, serum AFP decreased to 643 ng/mL, and 3 months later, had drastically decreased to 7 ng/mL. At the same time, a marked reduction in liver lesion size was observed by MRI, evaluated as a partial response according to Modified Response Evaluation Criteria in Solid Tumors (mRECIST) [19]. In August 2010, computer tomography (CT) scanning showed a single hypodense lesion of 13 mm in hepatic segment II without any other liver lesions, and that enlarged abdominal lymph nodes were stable and neoplastic thrombosis was not detected. Given the presence of a single lesion, the possibility of residual disease ablation was explored using hepatic contrast-enhanced ultrasound. Two suspicious lesions for HCC were detected in hepatic segments II and III, without a typical contrastographic appearance. The lesions were submitted to needle biopsy, and histological analysis identified a nodule of low-grade dysplasia in segment II and micro and macronodular cirrhosis with light activity in segment III. The patient underwent metronomic capecitabine until July 2013 when, considering the sustained CR, treatment was discontinued and the patient entered a follow-up program. The final MRI (February 2017, Fig. 1c, d) confirmed the CR. To date, the patient is alive and in good health.

### Case 2

A 66-year old man had multiple liver lesions involving approximately 70% of the right liver, multiple nodules in the left lobe, and a right portal thrombosis in the setting of non-alcoholic steatohepatitis (CT scan in August 2012, Fig. 2a, b). Positron emission tomography (PET) with 2-(fluorine-18)-fluoro-2-deoxy-d-glucose (FDG-PET) identified bone metastases in the proximal portion of the right femur, in the right ischial tuberosity, in the left acetabulum, in the left scapula, and in the third left costal arch. Moreover, a PET with (11)C-choline confirmed the hepatic and skeletal lesions and identified other metastases in the pelvic bones, rachis, and ribs. In October 2012, serum AFP was 1909 ng/mL. Considering the typical contrastographic pattern of the liver lesion by CT scanning and the elevated AFP level, a diagnosis of HCC was made according to European Association for the Study of the Liver (EASL) guidelines [20].

**Fig. 2:**
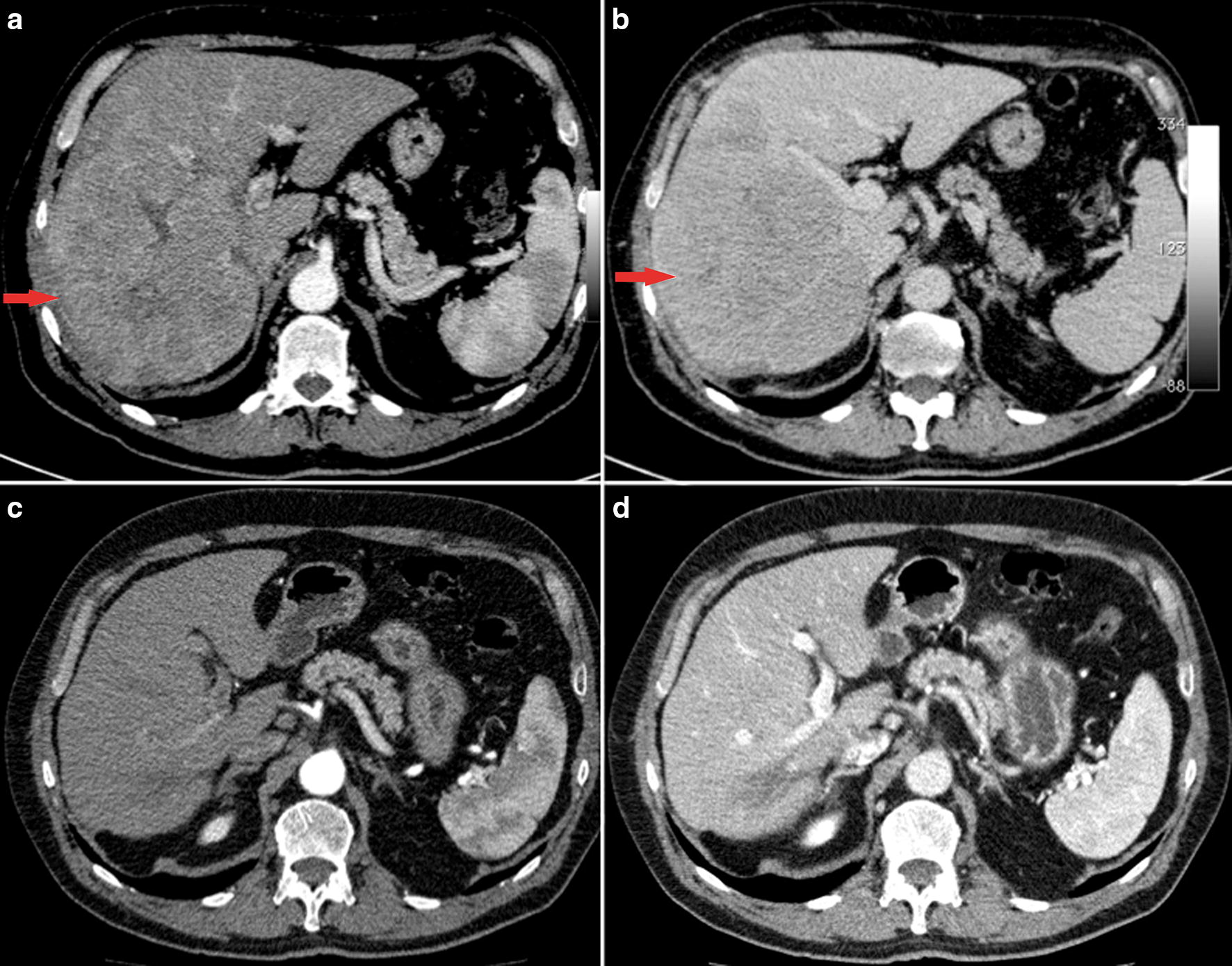
Lesions in case 2: axial CT images showing a large (10 cm) heterogeneous hypervascular hepatocellular carcinoma of segment VII in the arterial phase at baseline (**a**), with unequivocal wash-out in the venous phase (**b**); 32 months after starting systemic treatment with metronomic capecitabine, CT images show a complete response with the absence of viable tumour burden in the arterial phase (**c**), and only a 2 cm hypodense “scar” better identified in the venous phase (**d**)

In December 2012, the patient started systemic treatment with sorafenib 800 mg/bid. Ten days later, the treatment was discontinued because of G3 skin toxicity (Stevens–Johnson syndrome). In January 2013, the patient started metronomic capecitabine (500 mg twice daily, continuous administration), which was well-tolerated. In March 2013, a new CT scan showed a reduction in the number and size of the liver lesions with significant intralesional necrotic areas. Subsequent FDG-PET scanning (April 2013) showed the complete absence of pathological areas and, in parallel, AFP level had fallen to 3.3 ng/mL. In July 2013, a needle biopsy of the principal hepatic lesion evidenced fibrous connective tissue with histiocytic inflammation without tumour cells. An abdominal US scan (January 2014) revealed the presence of a single hypoechoic lesion of 1.4 × 1.3 cm. In December 2014, CT scanning showed a further size reduction of the hypodense hepatic lesion without vascular components, and it was therefore decided to discontinue treatment in the same month. CT and US images, supported by histological patterns, showed a necrotic lesion with gradual resolution (Fig. 2c, d). In the most recent US scan (September 2017), no hepatic lesion was detected and serum AFP was 3.6 ng/mL. The patient is currently alive and in excellent health.

### Case 3

**Fig. 3:**
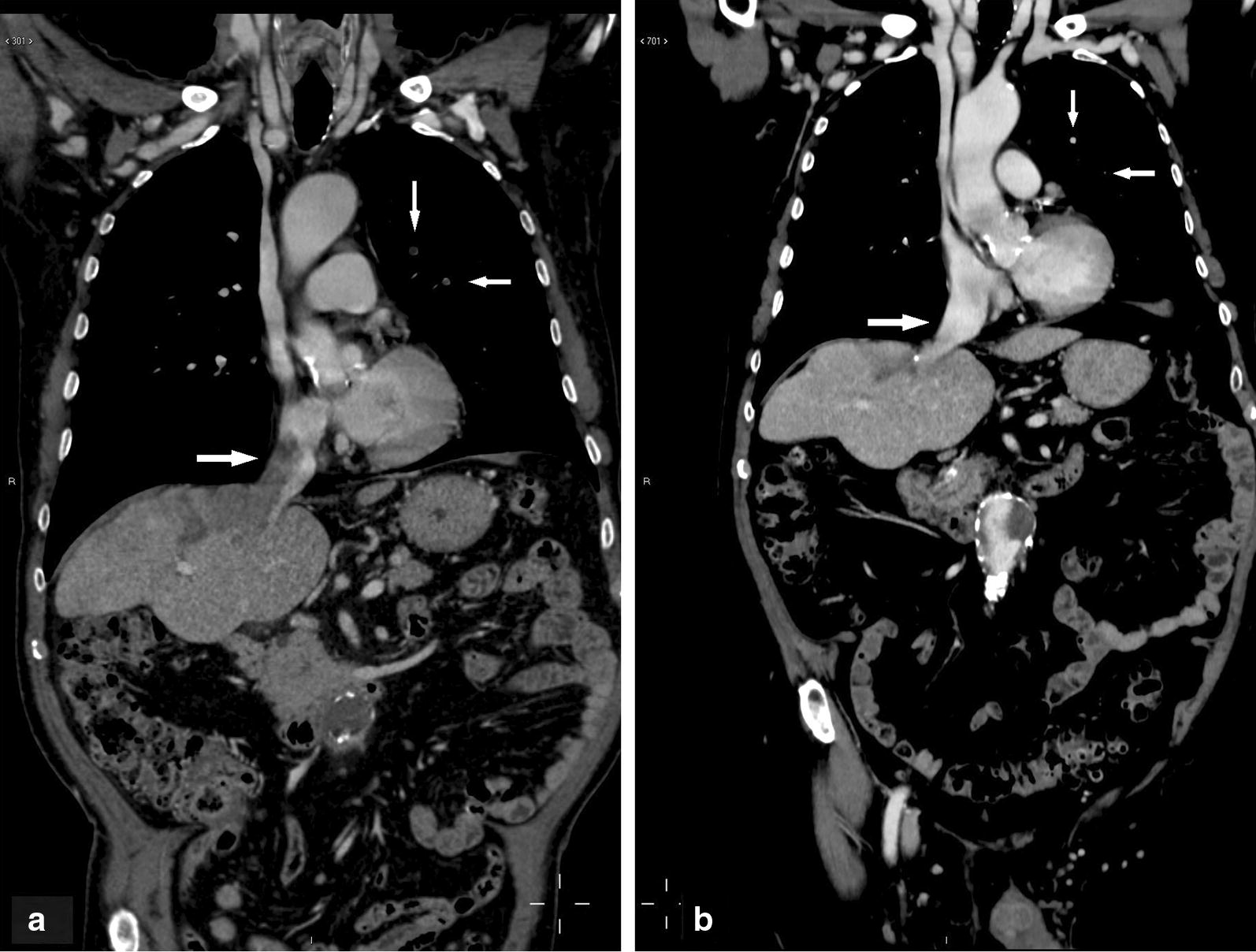
Lesions in case 3: **a** pre-treatment CT scan (coronal image of the portal fase). The image shows vascular invasion of the right and medium hepatic veins and of the inferior cava vein. Two neoplastic embolisms are present in the left lung. **b** Following 18 months of treatment with metronomic capecitabine, vascular vein invasion was resolved and pulmonary embolisms had disappeared

A 70-year-old man with hepatitis C virus cirrhosis was diagnosed with binodular HCC in Jul 2006 and treated with RFA and percutaneous ethanol injection (PEI). From March 2008 to March 2015, the patient experienced multiple tumour recurrences, which were managed using locoregional techniques (RFA, PEI, and one course of TACE). The patient came to our attention in October 2015 following HCC progression in the VIII segment associated with an invasion of the inferior vena cava and neoplastic pulmonary embolization. Serum AFP was 18,622 ng/mL. Systemic therapy with sorafenib was started at dosage of 400 mg/die, given the patient’s poor clinical condition, and increased to 600 mg/die after 10 days. The patient started experiencing severe fatigue, diarrhoea, and dizziness, which prompted a reduction in dosage to 400 mg/die in November 2015. In February 2016, following radiological progression (tumoural invasion of the right and median hepatic veins and enlargement of the neoplastic thrombus in the inferior cava vein) and a sharp increase in serum AFP (47,137 ng/mL), the patient was switched to capecitabine therapy (500 mg twice daily, continuous administration). CT scanning performed every 3 months showed the progressive reduction of pulmonary metastases, recanalization of the median hepatic vein, and progressive improvement in inferior cava vein invasion. Moreover, the tumour mass showed a complete devascularisation (Fig. 3a, b). Serum AFP levels decreased to 4583 ng/mL in May 2016, 5.5 ng/mL in September 2016, 2.5 ng/mL in November 2016, and 1.5 ng/mL in October 2017.

At the time of writing, the patient is in good clinical condition and continues to receive capecitabine treatment (500 mg bid), complaining only of modest fatigue.

## Discussion

The efficacy of metronomic capecitabine in advanced HCC has been evaluated both as frontline and post-sorafenib treatment in retrospective analysis [10, 12] and in a phase II prospective trial [9]. In the first retrospective study, the outcome of 26 patients treated with metronomic capecitabine after sorafenib failure was reported, with a median OS of 8 months [10]. Another retrospective case–control study reported a median OS of 12 months in patients treated with capecitabine versus 9 months in patients who received only BSC [21]. Furthermore, a large multicentre retrospective analysis compared patients treated with metronomic capecitabine or BSC after sorafenib discontinuation and found that the median survival was approximately 10 months in patients treated with metronomic capecitabine and less than 5 months in those receiving BSC. This benefit was confirmed in patients matched in a propensity analysis [22].

In 2013, our group published a phase II study on metronomic capecitabine in patients with advanced HCC. Metronomic capecitabine (500 mg twice daily continuously without breaks) was administered to 59 patients naïve to systemic therapy and 31 patients who were intolerant to sorafenib or following sorafenib failure. Median PFS and OS were 6.03 and 14.47 months, respectively, in the first-line group, and 3.24 and 9.77 months in the second-line group. Additionally, we recorded two cases of CR and one of partial response in sorafenib-naïve patients [9].

Other sustained (but incomplete) tumour responses have been observed in three cases of unresectable HCC treated with metronomic capecitabine [21, 23].

The difference between the absolute number of CR cases linked to sorafenib (51 cases of CR following a PubMed search with the string “HCC OR hepatocellular carcinoma AND sorafenib AND complete AND response”) and to metronomic capecitabine is likely attributable to the limited use of metronomic capecitabine for HCC, in contrast with the sorafenib, the standard treatment. No data on the duration of sorafenib-related CR are available in the literature, reinforcing the rationale for exploring the efficacy of metronomic capecitabine

Moreover, important positive aspects of metronomic capecitabine treatment include its approximately 100-fold lower cost than sorafenib, which is expected to further decrease in the near future following patent expiration. Savings to national health systems associated with the use of metronomic capecitabine are therefore likely to be significant compared with sorafenib treatment.

To identify a biomarker predictive of treatment response and, subsequently, patients with advanced HCC who are more likely to benefit from therapy at an optimal dose, it is necessary to explore the molecular determinants of susceptibility to a given treatment strategy. Variation in lactate dehydrogenase serum level during sorafenib treatment is an example of such a biomarker [24].

Further studies are needed to understand the phenomenon of the long-term response to metronomic capecitabine observed in this case report. We can speculate that tumours showing a dramatic response to treatment are more likely to display a specific molecular pattern, preventing drug resistance. The use of next-generation sequencing techniques may help to clarify the determinants of there markable responses observed in the cases reported here.

## Conclusion

The outcomes of the three cases of CR described here suggest that metronomic capecitabine represents a viable treatment option in patients with HCC and may be highly effective in a subgroup of “super respondent” patients, with a rare but possible CR. CR linked to sorafenib and metronomic capecitabine suggests that a specific subset of HCC maybe particularly responsive to antiangiogenic treatment.

## Abbreviations

HCC: hepatocellular carcinoma
CR: complete response
OS: overall survival
PFS: progression-free survival
TACE: transarterial chemoembolization
RFA: radiofrequency ablation
MRI: magnetic resonance imaging
AFP: alpha-fetoprotein
CT: computer tomography
CEUS: contrast enhanced ultrasound
FDG-PET: positron emission tomography (PET) with 2-(fluorine-18)-fluoro-2-deoxy-d-glucose
US: ultrasound
PEI: ethanol injection
5-FU: 5-fluorouracil
TSP-1: thrombospondin-1
VEGF: vascular endothelial growth factor
b-FGF: basic fibroblast growth factor
HIF-1: hypoxia-inducible factor-1
BSC: best supportive care
